# Supplementary material for: Transcriptional Expression of SLC2A3 and SDHA Predicts the Risk of Local Tumor Recurrence in Patients with Head and Neck Squamous Cell Carcinomas Treated Primarily with Radiotherapy or Chemoradiotherapy
Source: Int J Mol Sci. 2025 Mar 9;26(6):2451. doi: 10.3390/ijms26062451 (PMC11942407; doi:10.3390/ijms26062451)
Supplement: Supplementary file 1 [file ijms-26-02451-s001.zip › ijms-3475485-supplementary.pdf]

Supplementary Material

Figure S1. Distribution of SLC2A3 transcript expression of the primary location of the tumor previous to the treatment according to the local control after (chemo-)radiotherapy.

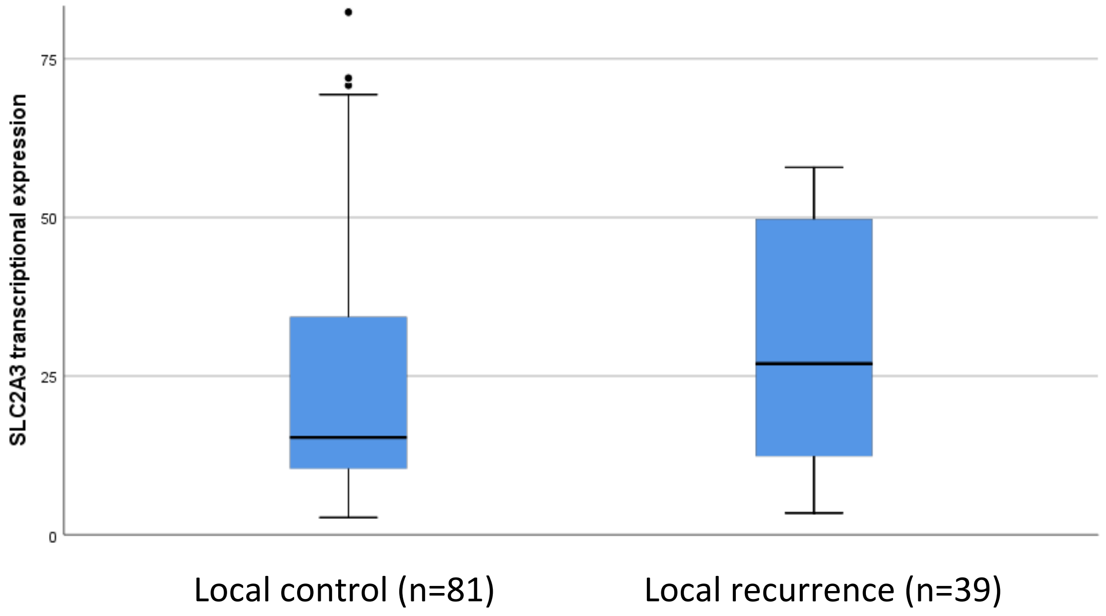

Figure S2. Local recurrence-free survival according to the transcriptional expression categories of SLC2A3, SLC16A3 and SDHA.

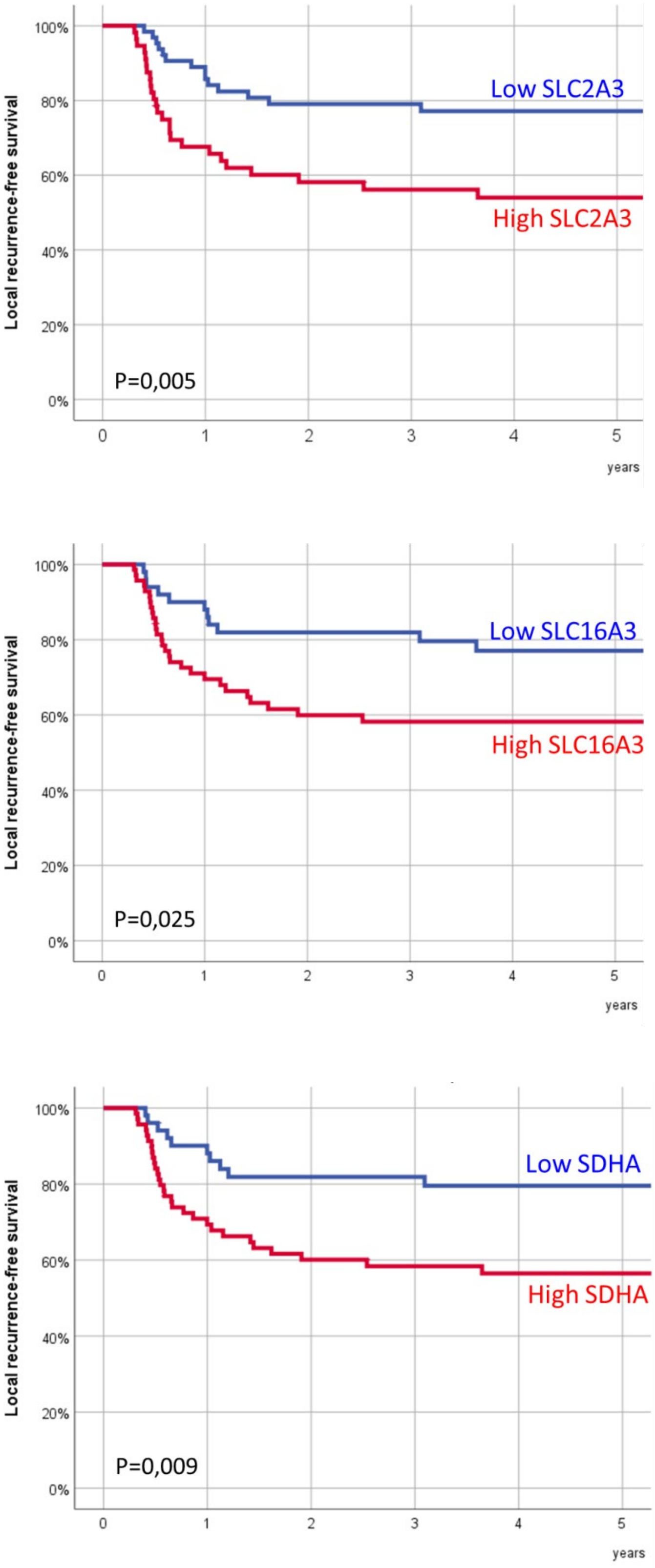

Figure S3. Local recurrence-free survival according to SLC2A3 and SDHA transcriptional expression depending on the type of treatment. Group 1, patients with low SLC2A3 expression, or patients with high SLC2A3 and low SDHA expression; Group 2, patients with high SLC2A3 and high SDHA expression.

Radiotherapy (n=54)

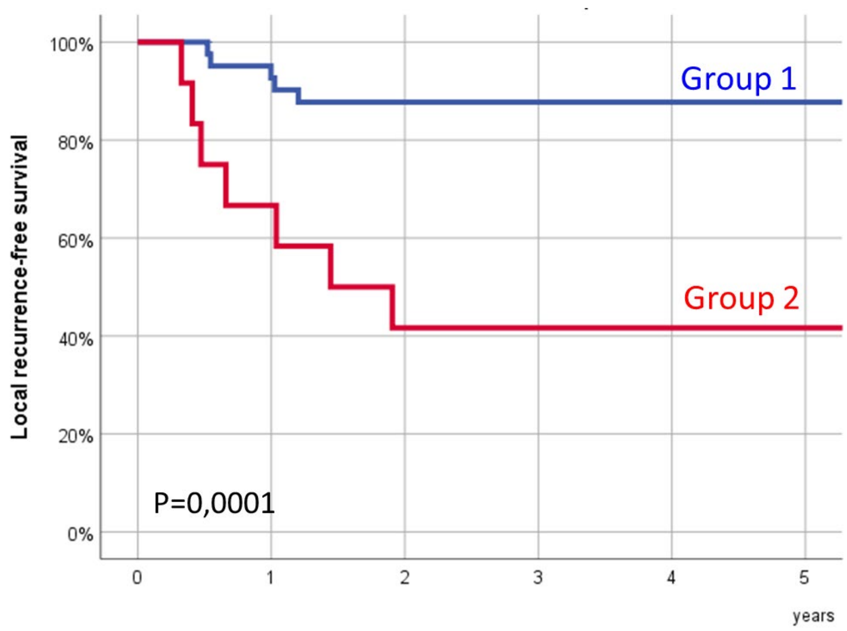

Chemoradiotherapy (n=66)

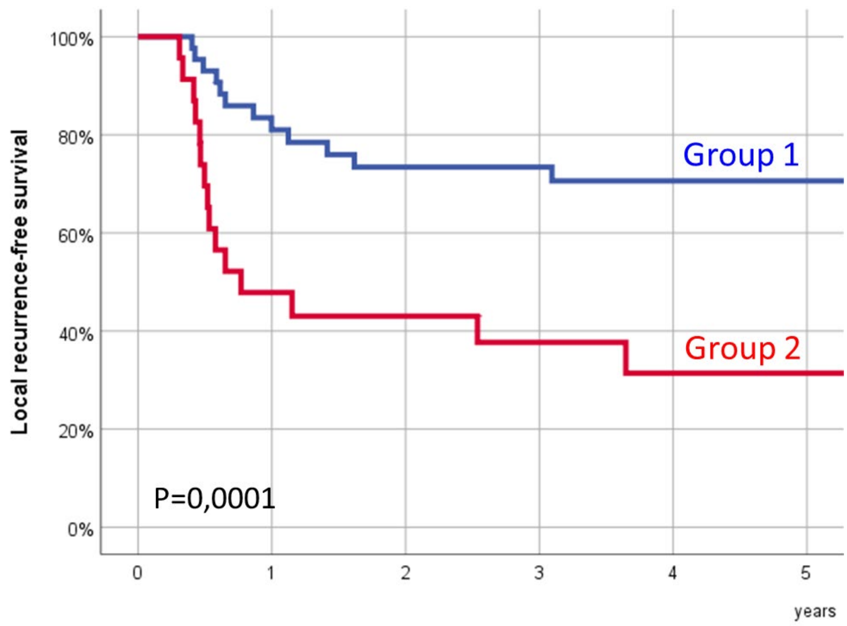

Figure S4. Local recurrence-free survival according to SLC2A3 and SDHA transcriptional expression depending on the local extension of the primary tumor. Group 1, patients with low SLC2A3 expression, or patients with high SLC2A3 and low SDHA expression; Group 2, patients with high SLC2A3 and high SDHA expression.

Early tumors (cT1-T2, n=70)

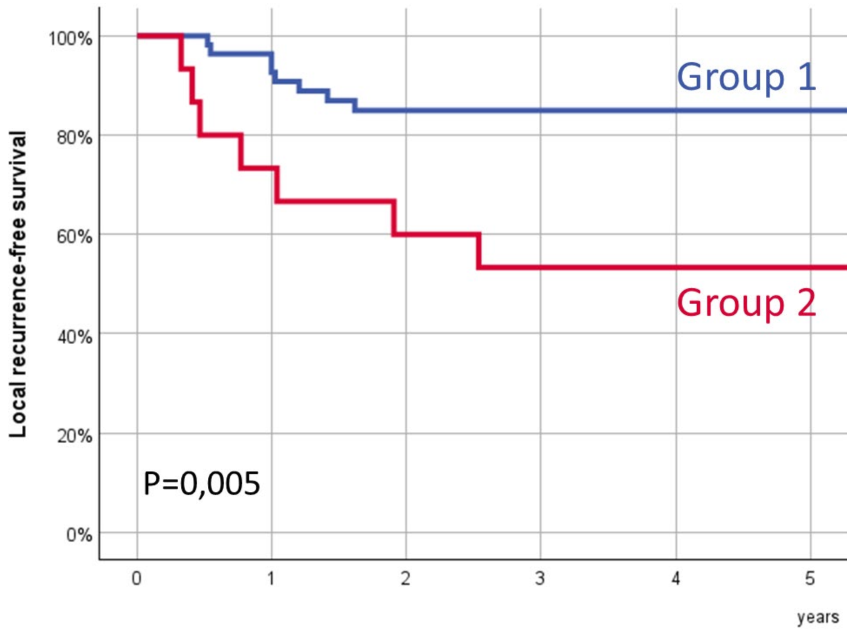

Advanced tumors (cT3-T4, n=50)

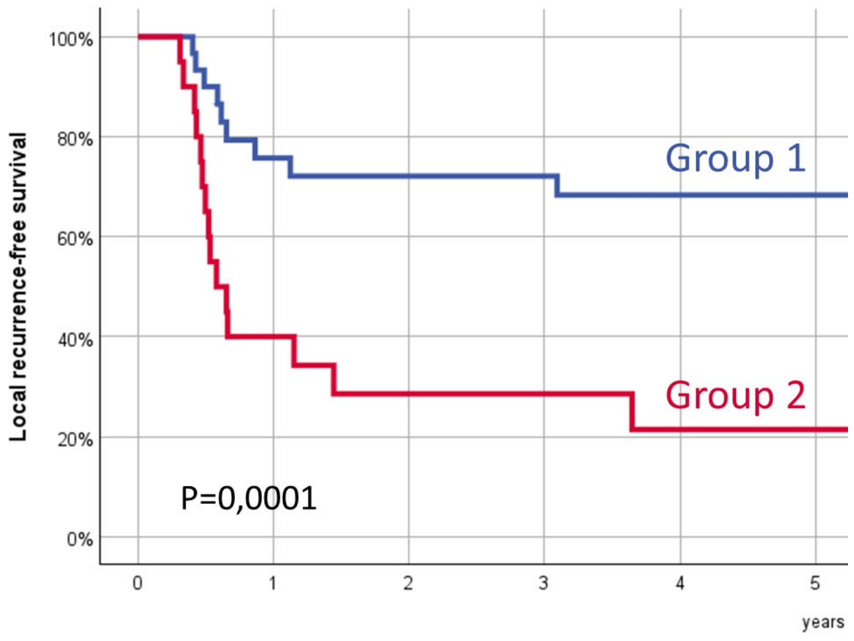

Figure S5. Local recurrence-free survival of patients with high and low SLC2A3 expression based on SDHA expression, and of patients with high and low SDHA expression based on SLC2A3 expression.

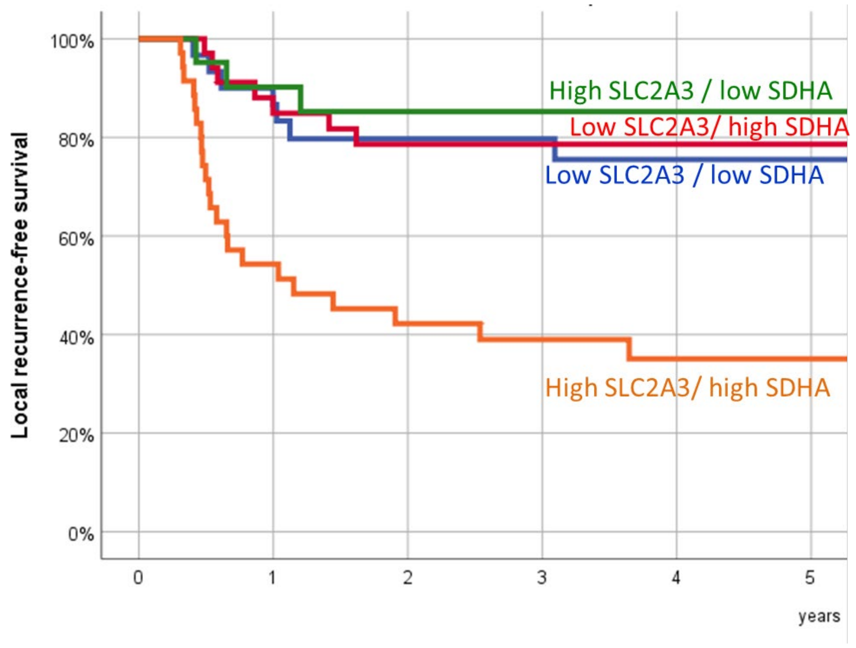

Figure S6. Distribution of SLC2A3 transcript expression of the primary location of the tumor of the TCGA patients according to tumor status.

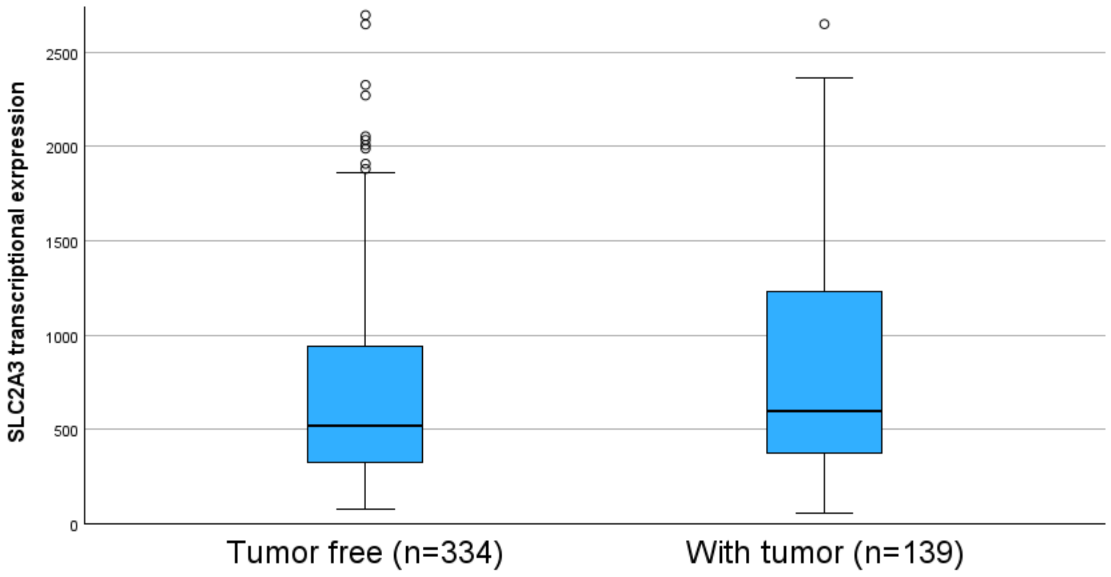

Figure S7. Classification and regression tree according to the transcriptional expression values of SLC2A3 and SDHA of the TCGA patients considering tumor status as the dependent variable.

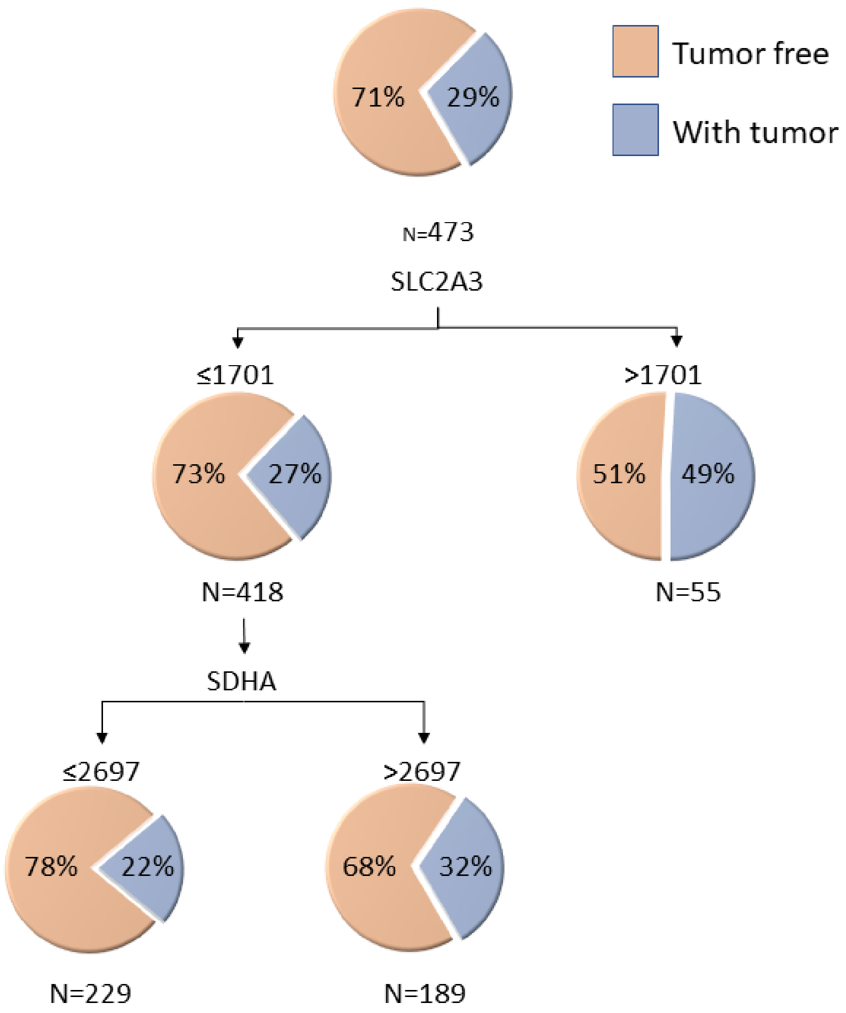

Table S1. Median value of the transcriptional expression of the genes analyzed according to the studied variables.

		SUCNR1	P	SLC2A3	P	SLC16A3	P	SDHA	P
**Sex**	Male	0.85	0.086	15.56	**0.011**	44.15	0.177	32.15	0.689
	Female	1.35		35.18		58.63		39.14	
**Toxics consumption**	No	2.40	0.073	28.56	0.425	53.85	0.109	32.50	0.867
	Moderate	0.87		12.61		40.07		32.23	
	Severe	0.83		18.59		46.48		32.15	
**Location**	Oral cavity	1.33	0.136	43.11	0.288	75.68	0.211	29.11	0.999
	Oropharynx	0.71		15.73		41.70		32.20	
	Hypopharynx	1.49		16.72		58.94		32.50	
	Larynx	0.84		17.69		43.78		32.13	
**Local extension**	cT1-2	0.86	0.970	15.40	0.066	46.48	0.132	30.57	0.551
	cT3-4	0.93		22.15		44.55		36.00	
**Regional extension**	cN0	0.87	0.599	17.69	0.723	43.78	0.723	31.29	0.434
	cN+	1.10		18.51		47.69		33.13	
**Stage**	I-II	0.87	0.970	16.37	0.066	43.93	0.132	28.88	0.551
	III-IV	0.87		19.16		47.60		32.81	
**Local recurrence**	No	0.85	0.525	15.32	**0.043**	42.24	0.217	29.03	0.085
	Yes	0.93		26.95		52.50		34.19	
**HPV***	Negative	0.63	**0.016**	15.64	0.223	43.52	0.883	32.18	0.698
	Positive	1.20		10.48		37.05		34.62	

* 42 patients with oropharyngeal carcinoma

Table S2. Distribution of patients based on SLC2A3-SDHA expression according to clinical variables.

		**Group 1**	**Group 2**	**P**
**Age (mean/standard deviation) years**		62.4/11.0	62.9/12.5	0.809
**Sex**	Male	79 (74.5%)	27 (25.5%)	0.025
	Female	6 (42.9%)	8 (57.1%)	
**Toxics consumption**	No	8 (61.5%)	5 (38.5%)	0.204
	Moderate	15 (88.2%)	2 (11.8%)	
	Severe	62 (68.9%)	28 (31.1%)	
**Location**	Oral cavity	3 (50.0%)	3 (50.0%)	0.630
	Oropharynx	37 (74.0%)	13 (26.0%)	
	Hypopharynx	10 (66.7%)	5 (33.3%)	
	Larynx	35 (71.4%)	14 (28.6%)	
**Local extension**	cT1-2	55 (78.6%)	15 (21.4%)	0.041
	cT3-4	30 (60.0%)	20 (40.0%)	
**Regional extension**	cN0	52 (73.2%)	19 (26.8%)	0.543
	cN+	33 (67.3%)	16 (32.7%)	
**Stage**	I-II	39 (81.3%)	9 (18.8%)	0.031
	III-IV	46 (63.9%)	26 (36.1%)	
**Histologic grade**	Well differentiated	9 (81.8%)	2 (18.2%)	0.702
	Moderately differentiated	68 (70.1%)	29 (29.9%)	
	Poorly differentiated	8 (66.7%)	4 (33.3%)	
**Treatment**	Radiotherapy	42 (77.8%)	12 (22.2%)	0.159
	Chemoradiotherapy	43 (65.2%)	23 (34.8%)	
**HPV***	Negative	23 (76.7%)	7 (23.3%)	0.997
	Positive	9 (75.0%)	3 (25.0%)	

* 42 patients with oropharyngeal carcinoma

Table S3. Characteristics of the patients of The Cancer Genome Atlas included in the validation study according to the tumor status.

		Tumor free	With tumor	P
Sex	Male	249 (70.3%)	105 (29.7%)	0.821
	Female	85 (71.4%)	34 (28.6%)	
Location	Oral cavity	194 (69.5%)	85 (30.5%)	0.700
	Oropharynx	56 (74.7%)	19 (25.3%)	
	Hypopharynx	6 (60.0%)	4 (40.0%)	
	Larynx	78 (71.6%)	31 (28.4%)	
Local extension	cT1	25 (78.1%)	7 (21.9%)	0.236*
	cT2	101 (74.3%)	35 (25.7%)	
	cT3	79 (64.2%)	44 (35.8%)	
	cT4	114 (68.7%)	52 (31.3%)	
	Unknown	15 (93.8%)	1 (6.3%)	
Regional extension	cN0	162 (72.3%)	62 (27.7%)	0.305*
	cN+	154 (67.5%)	74 (32.5%)	
	Unknown	18 (85.7%)	3 (14.3%)	

* Chi-square excluding unknown cases
